# Comparison of myocardial perfusion between the users of two antiepileptic medications: valproate vs. carbamazepine

**DOI:** 10.22038/AOJNMB.2023.67084.1465

**Published:** 2023

**Authors:** Zeinab Peymani, Abbas Tafakhori, Saeed Farzanehfar, Farnoosh Larti, Ali Hosseini, Mehrshad Abbasi

**Affiliations:** 1 Department of Nuclear Medicine, Shariati Hospital, Tehran University of Medical Sciences, Tehran, Iran; 2 Department of Neurology, School of Medicine, Imam Khomeini Hospital Complex, Tehran University of Medical Sciences, Tehran, Iran; 3 Department of Nuclear Medicine, Imam Khomeini Hospital Complex, Vali-Asr Hospital, Tehran University of Medical Sciences, Tehran, Iran; 4 Department of Cardiology, Imam Khomeini Hospital Complex, Tehran University of Medical Sciences, Tehran, Iran

**Keywords:** Valproate, Carbamazepine, Myocardial perfusion scan Epilepsy, Coronary artery disease A B S T R A C T

## Abstract

**Objective(s):**

The prevalence of coronary artery disease (CAD) is high in patients with epilepsy using antiepileptic drugs (AED). Epilepsy, AED, or the type and duration of AED use , may contribute to higher CAD risk.

In this study, myocardial perfusion imaging (MPI) was compared between patients using carbamazepine and valproate.

**Method:**

Out of 73 patients receiving carbamazepine or valproate monotherapy for more than 2 years, visited at a tertiary referral clinic, 32 patients participated in a 2-day stress and rest phases MPI. For each phase, 15-25 mCi 99mTc-MIBI was injected, at peak exercise or by pharmacologic stimulation for the stress phase. SPECT with cardiac gating was done by a dual-head gamma camera and processed and quantified. Scans with at least one definite reversible hypo-perfusion segment were considered abnormal.

**Results:**

Seventeen patients received carbamazepine monotherapy and 15 valproates. Age and duration of AED use were similar between the groups. Two scans were abnormal (6.3%) both in valproate group (13.3%). Duration of AED use was higher in patients with abnormal scans. In patients receiving monotherapy >2 years, the frequency of abnormal MPI was similar between groups (P-value=0.12). In patients receiving monotherapy > 5 years, prevalence of abnormal MPI was higher in the valproate group (28.6% vs. 0.0%; P-value=0.042). Considering valproate subgroup, ischemic patients had higher duration of AED use, comparing with the normal patients (17.0±4.2 vs. 6.4±4.8, P-value=0.014).

**Conclusion:**

MPIs were abnormal in patients receiving valproate after 5 years compared to patients receiving carbamazepine. Long-term valproate use may increase the risk of CAD.

## Introduction

 There have been several reports concerning the increased cardiac event risk in patients with epilepsy (1). Certain considerable epidemiological studies confirmed the increased prevalence of cardiovascular diseases in this population (2). We previously showed that the carotid intima-media thickness as a surrogate factor for coronary artery disease (CAD) was high in patients with epilepsy compared to the control population (3). Nevertheless, some pieces of evidence correlated the increased risk in these patients to the medications they consumed rather than their underlying disease or their inherited genetics (4). Dyslipidemia and atherogenesis causal relationship with phenytoin, valproate, and carbamazepine have previously been reported (5). Carbamazepine is a hepatic enzyme inducer drug. There are shreds of evidence to support that dyslipidemia (6) and microangiopathy (7) are more prevalent in patients receiving enzyme inducer medications, albeit the contribution of these medications to the atheroma formation is not generally accepted (8). On the other hand, enzyme inhibitor medications, including valproate, are also reported to increase the risk for CAD in some studies (2). The increased risk of CAD in both enzyme inducer and inhibitor medications sounds strange and one may return to the idea that the CAD risk is essentially high in patients with epilepsy. A major obstacle to assess the CAD risk in patients with epilepsy is that the endpoint assessment including coronary artery angiography is not indicated and necessary in the asymptomatic young population of patients with epilepsy. The next valid and precise modality to evaluate CAD is myocardial perfusion imaging (MPI). In the current study, we intended to compare the MPI in patients using valproate and carbamazepine for the treatment of epilepsy.

## Methods

 Seventy-three patients aging 16 to 50 years with a history of epilepsy receiving monotherapy with either valproate or carbamazepine for at least 2 years were asked to participate. Patients with a history of diabetes mellitus and hypertension were not included. Thirty-two patients consented and accepted to participate in two-day stress and rest phases MPI. The histories of epilepsy and medication use were collected. The stress was done by exercise tolerance test or in those not suitable for exercise test by slow infusion of dipyridamole or dobutamine. For the stress day, 20-25 mCi ^99m^Tc-MIBI (Pars isotope, Tehran, Iran) was injected at maximal exercise, 4 minutes after slow dipyridamole injection, or at optimal heart rate during dobutamine infusion. The imaging was done 30 to 90 minutes after stress. For the rest phase, 15-20 mCi ^99m^Tc MIBI was injected with imaging delay for 60-120 min. MPI was acquired by a dual-head gamma camera (Forte ADAC, Philips, Milpitas, CA, USA) with the following specification: projection time of 25 to 30 sec, 16 stops, matrix size of 64×64, and 8 frames cardiac cycle gating. The images were processed using AutoCount, and quantitative perfusion and gated SPECT analyses were performed and evaluated. The final interpretation of the scans was done by the consensus of two nuclear physicians having the access to the history of the patients concealing the name of anti-epileptic medication. If any time, 2 interpreters did not reach a consensus, the opinion of a third nuclear physician was acquired. The MPIs with any severity and extent of ischemia were considered abnormal.

## Results

 Thirty-two patients (11 females; 34.4%) aged 30.4±9.3 years were recruited. Twenty-eight patients did not report chest pain, 2 patients had non-angina chest pain (one in the valproate group) and 2 patients had atypical chest pain (one in the valproate group). Six patients were smokers (4 in the valproate group) and a patient had a history of dyslipidemia (in the carbamazepine group). Out of all, 15 patients in the valproate group and 17 patients in the carbamazepine group, had received their antiepileptic drugs (AED) monotherapy for at least 2 years before the study. The health characteristics of participants are presented in Table 1. Twenty-one patients (65.6%) performed the exercise tolerance test and 10 (31.2%) underwent pharmacological stress (9 with Dipyridamole and 1 with Dobutamine).

**Table 1:** Health characteristics and myocardial perfusion and gating quantification data

	** Valproate group**	** Carbamazepine group**	** Total**	** P-value**
**Sex**	3 F, 12 M	8 F, 9 M	11 F, 21 M	
**Age**	27.9±8.5	32.6±9.6	30.4±9.3	0.16
**BMI**	23.1±5.7	25.8±4.9	24.4±5.3	0.25
**LVEF**	62.5±6.2%	63.8±7.0%	63.2±6.6%	0.60
**SSS**	0.6±1	2.6±2.8	1.4±2.1	0.39
**SDS**	0.5±0.9	1.6±2.1	0.9±1.6	0.33
**EDV**	66.5±15.6	71.2±21.8	68.8±18.7	0.51
**TID**	1.0±0.1	1.0±0.1	1.0±0.1	0.77
**LHR**	0.3±0.1	0.4±0.1	0.3±0.1	0.58

LVEF, left ventricular ejection fraction; SSS, summed stress score; SDS, summed difference score; EDV, end diastolic volume; TID, transient ischemic dilation; and LHR, lung heart ratio

**Figure 1:**
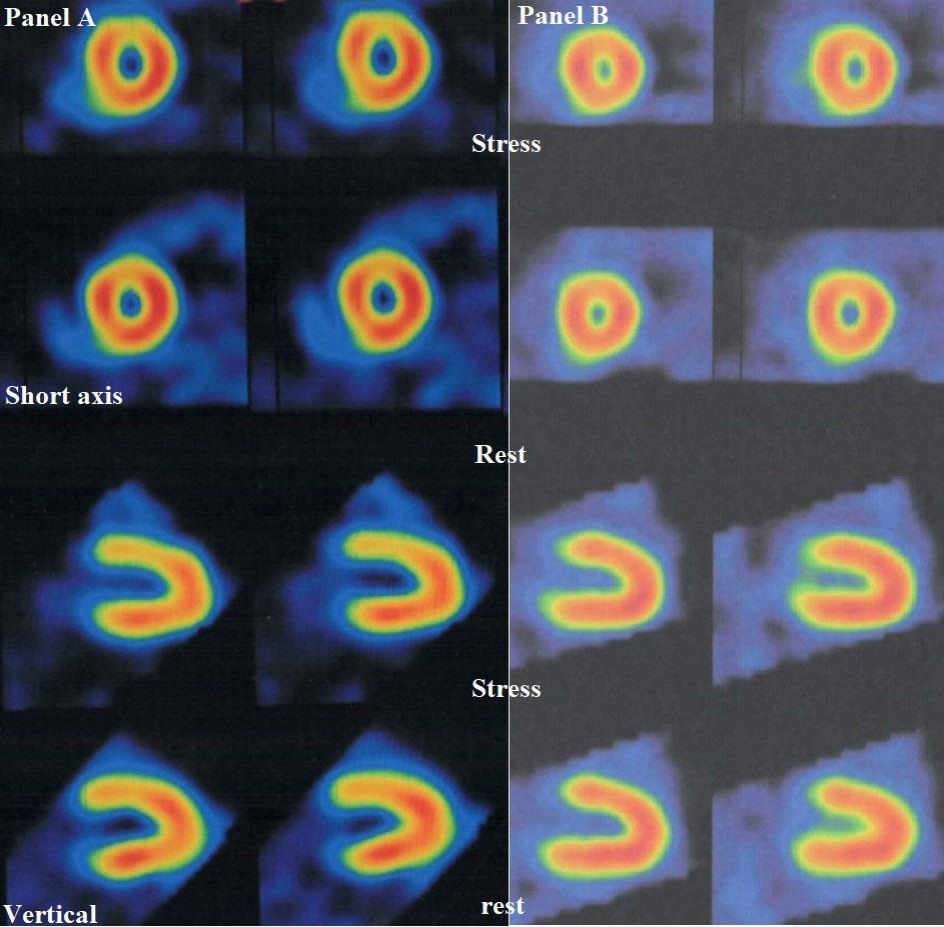
The myocardial perfusion scan of 2 patients with typical perfusion abnormality (19 years old male, 14 years of Valproate use; panel **A**) and typical normal scan (16 years old male; panel **B**)

**Figure 2:**
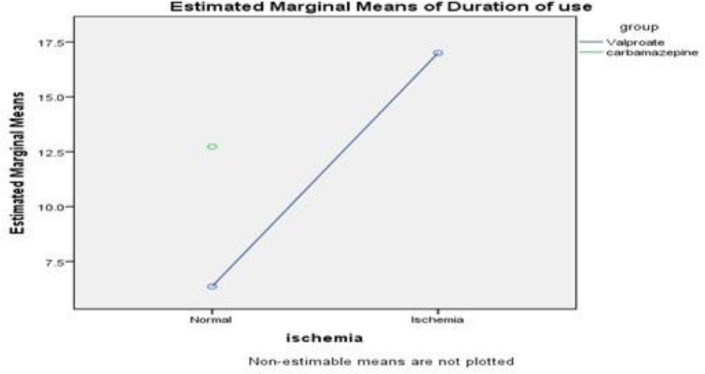
Estimated marginal means of durationof use

Two patients had abnormal MPI (Figure 1); both of them were using valproate (2 of 15: 13.3% of patients using valproate and 6.3% of total patients). In patients receiving monotherapy for at least 2 years, there was no significant difference for the prevalence of abnormal MPI between the two groups (P-value=0.12). Subgroup analysis demonstrated in a category of patients using medication for more than 5 years (n=20; 7 of them using valproate) the prevalence of abnormal MPI was higher in the valproate group (28.6% vs. 0.0%; P-value=0.042). The mean duration of Carbamazepine consumption was 12.7±7.1 years and the mean duration of Valproate use was 8.0±6.0, the differences showed no meaningful statistical significance (P-value=0.07; Figure 2). Considering both groups, the duration of medication use was not statistically correlated with the MPI result (ischemia vs. normal MPI, P-value=0.18). 

 Considering the Valproate subgroup, ischemic patients had meaningfully longer durations of drug consumption, comparing with the normal patients (17.0±4.2 vs. 6.4±4.8, P-value=0.014).

 The quantification indices of MPI were similar between patients on carbamazepine or valproate (Table 1). There was no remarkable difference between the quantification data regarding the ischemia, ventricular size, and ejection fraction. Mean Lung to hearty ratio (LHR) was normal 0.3±0.1; nevertheless, 4 patients revealed abnormally increased pulmonary activities, one of them had abnormal MPI (LHR>0.4).

## Discussion

 The prevalence of abnormal MPI at 6.3% was not high in the population of this study, patients with epilepsy. The prevalence of known CAD in the reference Iranian population is 7.7% (9). 

 Nevertheless, in the subgroup of patients using valproate, abnormal MPI was highly prevalent at 13.3%. This rate is considerable because it infers to the prevalence of unknown CAD while the patients with a known history of CAD or those with considerable risk factors i.e. hypertension and diabetes were not included. The distribution of the abnormal MPI was not different between the patients using carbamazepine and valproate monotherapy.

 Albeit, in the subpopulation of patients receiving the medication more than 5 years, the prevalence of abnormal MPI was higher in the patients using valproate. In fact, abnormal MPI in the current study was detected in patients who had used valproate for 14 and 20 years. The previous studies support the correlation of the accumulated AED dose or the duration of prescription with the occurrence of CAD (2, 5, 9, 10). Considering that the duration of carbamazepine use (12.7±7.1) was not less than that of valproate (8.0±6.0; p=0.71), the higher occurrence of myocardial ischemia after long use of valproate could be considered supporting evidence for a possible causal relationship between valproate use and myocardial ischemia. The concept of the association of longer valproate use and atherogenesis, increased carotid intima-media thickness, and dyslipidemia, has been documented previously (2, 5, 9-11). However, the current study is unique documenting a change of endpoint of the atherogenesis, MPI.

 There has been remarkable debate on the epidemiology and etiology of CAD in patients with epilepsy. It is documented that CAD and CAD-related mortality are higher in these patients (12-14). Albeit, the increased CAD risk is attributable to the side effects of AEDs. The data of patients with epilepsy not using AED is scant (15) hence the conclusion on whether epilepsy or AED causes higher CAD risk remains to be made. On the other hand, the studies provide controversial results on what AED, enzyme inducer or inhibitor, contributes more to atherogenesis. Carbamazepine is suspected to cause dyslipidemia due to hepatic enzyme induction and hyperhomocysteinemia (16-19). 

 Consequent increase of the activity of the P450 hepatic enzyme, which plays a major role in cholesterol metabolism, disturbs the lipid profile of the patient. Although valproate is an enzyme inhibitor, contrary to the belief of improving the lipid profile, it plays distinct atherogenic roles. Valproate-induced Carnitine depletion leads to decreased fatty acid metabolism (20) as well as causing insulin resistance (21). Valproate causes hyper homocysteinemia, non-HDLc hyperlipidemia, and high hs-CRP level correlating well with increased risk of atherogenesis (11, 19, 22-24). We did not assess the homocysteine and uric acid in the current study. Previously, we have shown the correlation of hyper homocysteinemia and abnormal MPI, correctable with folic acid (25). Measurement of homocysteine and uric acid levels in future studies or empiric treatment with folic acid or vitamin B supplements may provide further insight.

 Previously, we reported while carotid intima-media thickness (IMT) as a surrogate factor of CAD is higher in patients with epilepsy on AED for more than 2 years, there was no predilection of higher IMT for valproate or enzyme inducer AEDs including carbamazepine (3). In that study, the data of patients using monotherapy of carbamazepine and those patients using phenobarbital, phenytoin, and combination therapies were accumulated, hence reduced the comparability of the results for valproate and carbamazepine. 

 The current study suffers certain flaws; first, the measurement was not done for lipid profile, uric acid, homocysteine, and folic acid levels. We collected the history of dyslipidemia, but the measurements were not done for the study purpose. Such assessments would shed further light on the underlying mechanism valproate contributes to CAD. Second, exclusion of patients with a history of diabetes and hypertension reduced the power to extrapolate the results, as the AEDs are suspected to induce insulin resistance and also may dilute the reported prevalence of CAD in the studied population.

## Conclusion

 The prevalence of CAD in long-term valproate users is higher than their counterpart carbamazepine users. For the selection of AED for long-term use, possible future cardiac side effects should be considered. Regarding the predilection of CAD in patients with epilepsy, based on the correlation of valproate with abnormal MPI in the current study, one may infer that denoted high CAD prevalence in these patients is attributable to the therapy rather than the nature of the disease.

## Compliance with Ethical Standards

 This study has been approved by the Tehran University of Medical Sciences ethics committee and has therefore been performed in accordance with the ethical standards laid down in the 1964 Declaration of Helsinki and all subsequent revisions.

## Informed consent

 Informed consent was obtained from all individual participants included in the study.

## Conflict of interests

 The authors declare that they have no conflict of interest.

## Funding

The research was done as a part of the residency thesis of Tehran University of Medical Sciences.

## References

[1] Jansen K, Lagae L (2010). Cardiac changes in epilepsy. Seizure..

[2] LoPinto-Khoury C, Mintzer S (2010). Antiepileptic drugs and markers of vascular risk. Curr Treat Options Neurol..

[3] Mehrpour M, Shojaie M, Zamani B, Gharibzadeh S, Abbasi M (2014). Atherogenic consequence of antiepileptic drugs: a study of intima-media thickness. Neurol Sci..

[4] Lu B, Elliott JO (2012). Beyond seizures and medications: normal activity limitations, social support, and mental health in epilepsy. Epilepsia..

[5] Chuang YC, Chuang HY, Lin TK, Chang CC, Lu CH, Chang WN (2012). Effects of long‐term antiepileptic drug monotherapy on vascular risk factors and atherosclerosis. Epilepsia..

[6] Mintzer S (2010). Metabolic consequences of antiepileptic drugs. Curr Opin Neurol..

[7] Chen NC, Chen CH, Lin TK, Chen SD, Tsai MH, Chang CC (2018). Risk of Micro angiopathy in Patients with epilepsy under long-term antiepileptic Drug Therapy. Front Neurol..

[8] Keenan N, Sadlier LG, Wiltshire E (2014). Vascular function and risk factors in children with epilepsy: associations with sodium valproate and carbamazepine. Epilepsy Res..

[9] Meysamie A, Salarvand F, Khorasanizadeh M, Ghalehtaki R, Eskian M, Ghodsi S (2017). Cardiovascular risk assessment by FRS and SCORE in Iranian adult population. J Diabetes Metab Disord..

[10] Lai Q, Shen C, Zheng Y, Zhang Y, Guo Y, Ding M (2017). Effects of antiepileptic drugs on the carotid artery intima-media thickness in epileptic patients. J Clin Neurol..

[11] Talaat FM, Kamel T, Rabah AM, Ahmed SM, El-Jaafary SI, Abdelaziz GH (2015). Epilepsy and antiepileptic drugs: risk factors for atherosclerosis. Int J Neurosci..

[12] Neligan A, Shorvon SD (2010). Frequency and prognosis of convulsive status epilepticus of different causes: a systematic review. Arch Neurol..

[13] Saleh D, Ismail MA, Ibrahim AM (2012). Non-alcoholic fatty liver disease, insulin resistance, dyslipidemia and atherogenic ratios in epileptic children and adolescents on long term antiepileptic drug therapy. Pak J Biol Sci..

[14] Sankhyan N, Gulati S, Hari S, Kabra M, Ramakrishnan L, Kalra V (2013). Noninvasive screening for preclinical atherosclerosis in children on phenytoin or carbamazepine monotherapy: a cross sectional study. Epilepsy Res..

[15] Hamed SA, Hamed EA, Hamdy R, Nabeshima T (2007). Vascular risk factors and oxidative stress as independent predictors of asymptomatic atherosclerosis in adult patients with epilepsy. Epilepsy Res..

[16] Shakirullah Shakir NA, Nazish H (2018). Carbamazepine Verses Valproic Acid as Monotherapy in Epileptic Patients. J Coll Physicians Surg Pak..

[17] Bano S, Zuberi NA, Alam SM (2017). Correlation between Hyper homocysteinemia and Common Carotid Artery Intima media Thickness in Carbamazepine treated Epileptic patients using Ultrasonography. Pak J Med Sci..

[18] Vyas MV, Davidson BA, Escalaya L, Costella J, Saposnik G, Burneo JG (2015). Antiepileptic drug use for treatment of epilepsy and dyslipidemia: systematic review. Epilepsy Res..

[19] Yamamoto Y, Terada K, Takahashi Y, Imai K, Kagawa Y, Inoue Y (2016). Influence of anti-epileptic drugs on serum lipid levels in adult epilepsy patients. Epilepsy Res..

[20] Jallon P, Picard F (2001). Bodyweight gain and anticonvulsants. Drug Saf..

[21] Pylvänen V, Pakarinen A, Knip M, Isojärvi J (2006). Insulin-related metabolic changes during treatment with valproate in patients with epilepsy. Epilepsy Behav..

[22] Sharma TK, Vardey S, Sitaraman S (2015). Evaluate the Effect of Valproate Monotherapy on the Serum Homocysteine, Folate and Vitamin B12 Levels in Epileptic Children. Clin Lab..

[23] Ni G, Qin J, Fang Z, Chen Y, Chen Z, Zhou J, Zhou L (2014). Increased homocysteine levels in valproate-treated patients with epilepsy: a meta-analysis. BMJ open..

[24] Gorjipour F, Asadi Y, Osguei NK, Effatkhah M, Samadikuchaksaraei A (2013). Serum level of homocysteine, folate and vitamin-B12 in epileptic patients under carbamazepine and sodium valproate treatment: a systematic review and meta-analysis. Iran Red Crescent Med J..

[25] Emami-Ardekani A, Esteghamati A, Farzanefar S, Abousaidi M, Abbasi M, Abdollahi S (2015). Folate therapy improves the stress-to-rest mean LV volume ratio in myocardial perfusion imaging in patients with diabetes. Ann Nucl Med..

